# Clinical advantage and outcomes of computed tomography‐based transvaginal hybrid brachytherapy performed only by sedation without general or saddle block anesthesia

**DOI:** 10.1002/cnr2.1607

**Published:** 2022-03-01

**Authors:** Noriyuki Okonogi, Kazutoshi Murata, Toshiaki Matsui, Yuma Iwai, Yasumasa Mori, Takashi Kaneko, Masaru Wakatsuki, Hiroshi Tsuji

**Affiliations:** ^1^ QST Hospital, National Institutes for Quantum Science and Technology Chiba Japan

**Keywords:** brachytherapy, cervical cancer, hybrid brachytherapy, interstitial brachytherapy, intracavitary brachytherapy, radiotherapy

## Abstract

**Background:**

Three‐dimensional image‐guided brachytherapy is the standard of care in cervical cancer radiotherapy. In addition, the usefulness of the so‐called “hybrid brachytherapy (HBT)” has been reported, which involves the addition of needle applicators to conventional intracavitary brachytherapy for interstitial irradiation.

**Aim:**

To evaluate the clinical outcomes of CT‐based HBT consisting of transvaginal insertion of needle applicators (CT‐based transvaginal HBT) and only intravenous sedation without general or saddle block anesthesia.

**Methods and results:**

This is a retrospective chart review of patients who received definitive radiotherapy, including CT‐based transvaginal HBT, between February 2012 and July 2019. The inclusion criteria were as follows: (i) histologically diagnosed disease, (ii) untreated cervical cancer, (iii) International Federation of Gynecology and Obstetrics (FIGO) stage IB1–IVA disease in the 2008 FIGO staging system, and (iv) patients who underwent CT‐based transvaginal HBT at least once in a series of intracavitary brachytherapy. Overall, 54 patients fulfilled the eligibility criteria in the present study. The median follow‐up period was 32 (IQR, 19–44) months. No patient complained of symptoms such as persistent bleeding or abdominal pain after the treatment. The 3‐year local control (LC), disease‐free survival, and overall survival rates for all 54 patients were 86.6%, 60.3%, and 90.7% (95% CI [81.3%–100.0%]), respectively. The 3‐year LC rate was 87.7% in patients with FIGO III–IVA and 90.4% in tumor size >6.0 cm. The incidence rate of late adverse events, grade ≥3, in the rectum and bladder was 0% and 1.8%, respectively. In the dose‐volume histogram analyses, transvaginal HBT increased the dose of HR‐CTV_D90_ by ~7.5% without significantly increasing the dose of organs at risk.

**Conclusion:**

Considering the favorable clinical outcomes, CT‐based transvaginal HBT may be a good option for treating cervical cancer.

## 1 INTRODUCTION

Uterine cervical cancer is one of the most common cancers in women worldwide.^1^ Radiotherapy (RT) plays a crucial role as a definitive treatment for patients with stage IB–IVA cervical cancer. Several randomized phase III studies and meta‐analyses have established the use of platinum‐based concurrent chemoradiotherapy (CCRT) as the standard of care for patients with stage IB–IVA cervical cancer.^2^, ^3^, ^4^

Intracavitary brachytherapy is a pivotal treatment for all patients with cervical cancer receiving RT or CCRT.^5^ In 2005, the Groupe Européen de Curiethérapie and the European Society for Radiotherapy & Oncology Gynecology proposed the concept of a three‐dimensional image‐guided brachytherapy (3D‐IGBT).^6^, ^7^ These studies provided the core concepts and defined the terms that would be used in 3D‐IGBT.^6^, ^7^ Many studies have demonstrated the relationship between local control (LC) probability and dose to the high‐risk clinical target volume (HR‐CTV) since these concepts were first introduced.^8^, ^9^, ^10^ The transition from traditional brachytherapy to 3D‐IGBT has led to improved LC rates and reduced late toxicity.^8^, ^9^, ^10^ More recently, EMBRACE‐I, a prospective multicenter study that employed magnetic resonance imaging (MRI)‐guided 3D‐IGBT for cervical cancer, has been published.^11^ In this study, the actuarial overall 5‐year LC rate was 92%, with limited severe toxicity in normal organs.^11^

MRI is undoubtedly the ideal imaging modality for 3D‐IGBT, owing to its superior visualization in soft tissue compared to computed tomography (CT).^12^ However, its availability for 3D‐IGBT is still limited in clinical settings. A recent nationwide survey of 3D‐IGBT in Japan showed that only 4% of 3D‐IGBT was performed using MRI.^13^ Similar surveys conducted in the US and Canada report limited use of MRI‐based 3D‐IGBT (34%–57%).^14^, ^15^ Notably, ~90% of newly diagnosed cervical cancer occur in low‐ to middle‐income countries, where access to MRI‐based 3D‐IGBT is difficult.^16^ Establishing a beneficial and easily accessible treatment strategy that replaces MRI‐based 3D‐IGBT is, therefore, crucial to improving treatment outcomes for a larger number of patients with cervical cancer. CT‐based 3D‐IGBT presents as a viable alternative. Several guidelines or protocols on HR‐CTV contouring for CT‐based IGBT have been reported hitherto,^17^, ^18^ and an international recommendation for CT‐based 3D‐IGBT has been recently published.^19^ With careful consideration of target volume contouring, the LC rates of these studies consisting of CT‐based 3D‐IGBT are comparable to those of MRI‐based IGBT.^10^, ^20^, ^21^

For irregularly shaped and/or bulky cervical cancer, the interstitial approach may be an effective treatment method. However, recent National Comprehensive Cancer Network guidelines mention that such interstitial brachytherapy should only be performed by individuals and institutions with appropriate experience and expertise.^22^ As a simplified approach, the usefulness of the so‐called “hybrid brachytherapy (HBT),” in which needle applicators for interstitial irradiation are added to conventional intracavitary brachytherapy, has been reported.^23^, ^24^ Recently, Murakami et al. reported the initial outcomes of CT‐based HBT for locally advanced cervical cancer.^24^ In this study, additional interstitial needle catheters were inserted perineally or vaginally under transrectal ultrasound guidance using saddle block anesthesia or local anesthesia and intravenous sedation.^25^ Although this method is simpler and easier to use than the interstitial approach, it still requires specialized skills and knowledge about the saddle block anesthesia procedure for perineal needle insertion.

Tan et al. proposed a method of HBT in an environment with limited medical resources, such as outpatient set‐ups.^26^ Although they did not report the oncological outcomes, they reported the feasibility of a combination of oxycodone 5 mg capsules, midazolam, and a paracervical block. As a further simplified approach, we have been performing CT‐based HBT consisting of transvaginal insertion of needle applicators (CT‐based transvaginal HBT) and only intravenous sedation without general or saddle block anesthesia. Here, we report the clinical outcomes, including the safety of CT‐based transvaginal HBT, in patients with cervical cancer in our institution.

## 2 METHODS

### 2.1 Patient eligibility

We retrospectively reviewed clinical outcomes in consecutive patients with cervical cancer who were treated with definitive RT/CCRT, including CT‐based transvaginal HBT, between February 2012 and July 2019 in our hospital. The inclusion criteria were as follows: (i) histologically diagnosed disease, (ii) untreated cervical cancer, (iii) International Federation of Gynecology and Obstetrics (FIGO) stage IB1–IVA disease in the 2008 FIGO staging system, and (iv) patients who underwent CT‐based transvaginal HBT at least once in a series of intracavitary brachytherapy. The Ethical Review Board committee of our institution approved this study (QST 20‐043, approved on March 11, 2021).

### 2.2 External beam radiotherapy and chemotherapy

External beam radiotherapy (EBRT) involves a combination of whole pelvic (WP) irradiation, and central shielding (CS), similar to traditional methods in Japan and parts of Asia.^27^, ^28^ CS has been used as a part of EBRT in anteroposterior/posteroanterior fields to lower the irradiation dose to the bladder and rectum. In brief, up to ~50 Gy radiation was delivered to the WP and pelvis sidewall, with a daily fraction dose of 1.8 or 2.0 Gy using 10 megavolt X‐rays. After 20, 30, or 40 Gy of WP‐EBRT, a 3‐cm wide CS was inserted. Boost EBRT of 6–10 Gy in 3–5 fractions were performed for patients with pelvic nodal metastasis. For patients with para‐aortic lymph node (PAN) metastases, 40 Gy of prophylactic EBRT to the para‐aortic lymph node region was performed, followed by 16–18 Gy in 8–9 fractions of boost EBRT to the metastatic PANs. Weekly cisplatin (40 mg/m^2^, up to five courses) was concurrently administered with EBRT. Patients older than 70 years or with severe concomitant diseases, including renal dysfunction, ischemic heart disease, or severe diabetes, did not receive chemotherapy.

### 2.3 Brachytherapy

Intracavitary brachytherapy using a ^192^Ir remote after loading system (microSelectron, Nucletron, Veenendaal, the Netherlands) was performed weekly. Three to five (an average of four) fractions of brachytherapy were administered after starting CS irradiation. A set of Fletcher‐Suit Asian‐Pacific applicators were used for the majority of patients. A tandem‐vaginal cylinder applicator was used for some patients with tumor infiltration into the lower vagina, or those who had a narrow vagina. Additional interstitial catheters (Trocar Point needles, 1.5‐mm φ, Nucletron; Elekta, Stockholm, Sweden) were inserted transvaginally into the tumor during HBT. Intravenous sedation using flunitrazepam was performed before the applicator insertion. The details of pain relief and sedation in our hospital are shown in Data S1. All applicator insertions were performed freehand under transabdominal ultrasound guidance.

After applicator implantation, CT data were acquired with the patient in the supine position. The CT slice thickness was 3 mm, and CT‐based treatment planning was performed. HR‐CTV contouring was performed with the same delineations as the Japanese Radiation Oncology Study Group recommendations.^17^ The findings of the gynecological examinations performed at diagnosis, brachytherapy, and those of MRI examinations performed at diagnosis and just before the first brachytherapy session were used as references.

### 2.4 Indications for CT‐based transvaginal HBT

The transvaginal HBT in our hospital was implemented in cases with one or more of the following criteria: (i) the tumor extended to the pelvic wall on gynecological examination/MRI findings prior to intracavitary irradiation, (ii) tumor remained unevenly distributed on the bladder or rectum side, (iii) ellipticity (ratio of the shortest distance to the longest distance from the center of the uterine lumen to the edge of the tumor on the MRI axial image) was greater than 2, and (iv) (at second or later brachytherapy) insufficient dose or overdose of HR‐CTV in organs at risk (OARs) in the previous brachytherapy. Ellipticity was assessed using MRI before the first brachytherapy (Data S2). HBT was not performed if the patient took anticoagulants. The same criteria were consistently used to evaluate all patients who were included in the present study.

### 2.5 Dose‐volume histogram parameters

We estimated the composited dose to HR‐CTV_D90_ and OARs D_2cc_ (the minimum dose delivered to the highest irradiated 2‐cc region). The cumulative EBRT and brachytherapy doses were summarized and normalized to a biological equivalent dose of 2 Gy per fraction (EQD_2_) using a linear‐quadratic model with an alpha/beta of 10 Gy for the HR‐CTV and 3 Gy for the OARs. The doses of pelvic irradiation with CS were not added to the EQD_2_, as was done in previous studies.^10^, ^19^, ^20^

### 2.6 Dose prescription and optimization of HBT

**FIGURE 1:**
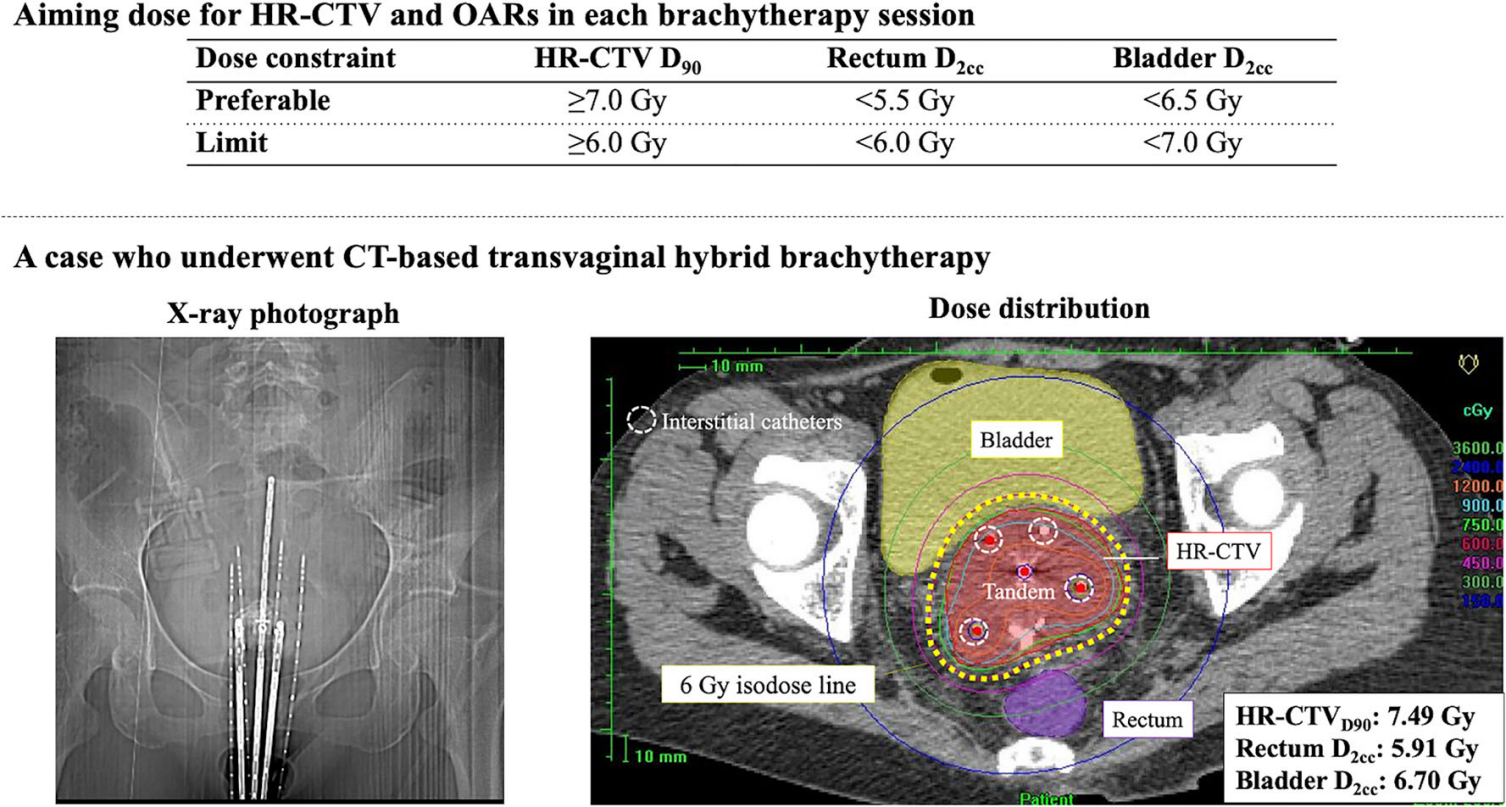
Aiming dose for HR‐CTV or OARs in each brachytherapy session and actual case who underwent CT‐based transvaginal hybrid brachytherapy. HR‐CTV, high‐risk clinical target volume; OARs, organs at risk

All treatment plans were formulated by the Oncentra planning system (Nucletron, Veenendaal, the Netherlands). The initial plan for each patient was generated based on Point A prescription. Dose adaptation by changing the dose at Point A was performed so that a dose >6 Gy was delivered to HR‐CTV_D90_. Thereafter, the dwell time allocation, including interstitial catheters, was modified. Finally, dose distribution was fine‐tuned using “graphical optimization” function. The aiming dose for HR‐CTV and OARs in each brachytherapy session in our institution and an actual session of CT‐based transvaginal HBT are shown in Figure 1. In each brachytherapy session, the doses aimed for HR‐CTV_D90_, Rectum D_2cc_, and Bladder D_2cc_ were ≥7.0, <5.5, and <6.5 Gy, respectively. There was no change in the prescription dose for each stage.

### 2.7 Follow‐up and evaluation for clinical outcomes

All patients were carefully monitored after treatment until they awakened and confirmed symptoms such as pain or bleeding after awakening. Patients' follow‐ups were scheduled every 1–3 months for the first 2 years and every 3–6 months thereafter. Gynecological examinations and imaging evaluations, including CT and MRI, were performed regularly. CT was taken once at the end of treatment, and every 6 months thereafter for the first 2 years. MRIs were taken 1 and 3 months after treatment, and every 6 months thereafter for the first 2 years. After which, CT and MRI were each taken annually. A tumor biopsy was performed for confirmation in cases of suspected local recurrence. Late toxicities in the present study were defined as any toxicity occurring 6 months after the initiation of RT. The grades of late toxicities were assessed in accordance with the toxicity criteria of the Radiation Therapy Oncology Group and the European Organization for Research and Treatment of Cancer system.^29^

### 2.8 Statistical analysis

The LC, disease‐free survival (DFS), overall survival (OS), and cumulative occurrence rates of late toxicity were evaluated using the Kaplan–Meier method. The LC and survival durations were calculated from the initiation of treatment. The log‐rank test was used for univariate analysis. The clinical factors in the two groups were compared using the Mann–Whitney *U* test. Statistical significance was set a*t p* < .05, and all statistical tests were two‐sided. Statistical calculations were performed using the IBM SPSS Statistics 27 software (IBM, Armonk, NY, United States).

## 3 RESULTS

### 3.1 Patient and treatment characteristics

The patient and treatment characteristics are summarized in Table 1. A total of 54 patients met the eligibility criteria in the present study. The median patient age was 65 (Interquartile range [IQR], 53–74) years, and the median follow‐up period was 32 (IQR, 19–44) months. Regarding histological subtypes, 45 patients had squamous cell carcinoma (Sq), and nine had adenocarcinoma or adenosquamous carcinoma (Ad/Adsq). The median tumor size at diagnosis was 6.0 (IQR, 4.9–6.8) cm, and 39 patients received concurrent chemotherapy. The median value of the ellipticity, which is one of the inclusion criteria of this study, was 2.07 (IRQ, 1.63–2.75). All patients underwent CT‐based IGBT; 51 patients underwent four sessions of CT‐based IGBT, two underwent three sessions of CT‐based IGBT, and one underwent five sessions of CT‐based IGBT. Of the 54 patients, 37 (68.5%) underwent three or more sessions of CT‐based transvaginal HBT.

**TABLE 1:** Patients and treatment characteristics

Characteristics	*n* = 54
Age, years	65 (53–74)
Follow‐up period, months	32 (19–44)
FIGO stage (2008)	
IB	2 (3.7%)
II	24 (44.4%)
III	21 (38.9%)
IVA	7 (13.0%)
Histological subtypes	
Squamous cell carcinoma	45 (83.3%)
Adenocarcinoma/adenosquamous carcinoma	9 (16.7%)
Pelvic LN metastasis	
No	23 (42.6%)
Yes	31 (57.4%)
Para‐aortic LN metastasis	
No	42 (77.8%)
Yes	12 (22.2%)
Tumor size, cm	6.0 (4.9–6.8)
Weekly cisplatin administrations	
No	15 (27.8%)
Yes	39 (72.2%)
EBRT, EQD2	
Whole pelvic irradiation (median), Gy	30 (20–40)
Central shielding irradiation (median), Gy	20 (20–30)
Number of HBT sessions	
1 session	9 (16.7%)
2 sessions	8 (14.8%)
3 sessions	15 (27.8%)
4 sessions	21 (38.9%)
5 sessions	1 (1.8%)

*Note*: Data except EBRT are median (IQR, interquartile range) or *n* (%), EBRT is median (range).

Abbreviations: EBRT, External beam radiotherapy; EQD2, equivalent dose of 2 Gy per fraction; FIGO, International Federation of Gynecology and Obstetrics; HBT, hybrid brachytherapy; LN, lymph node; NOS, not otherwise specified.

### 3.2 Clinical outcomes

**FIGURE 2:**
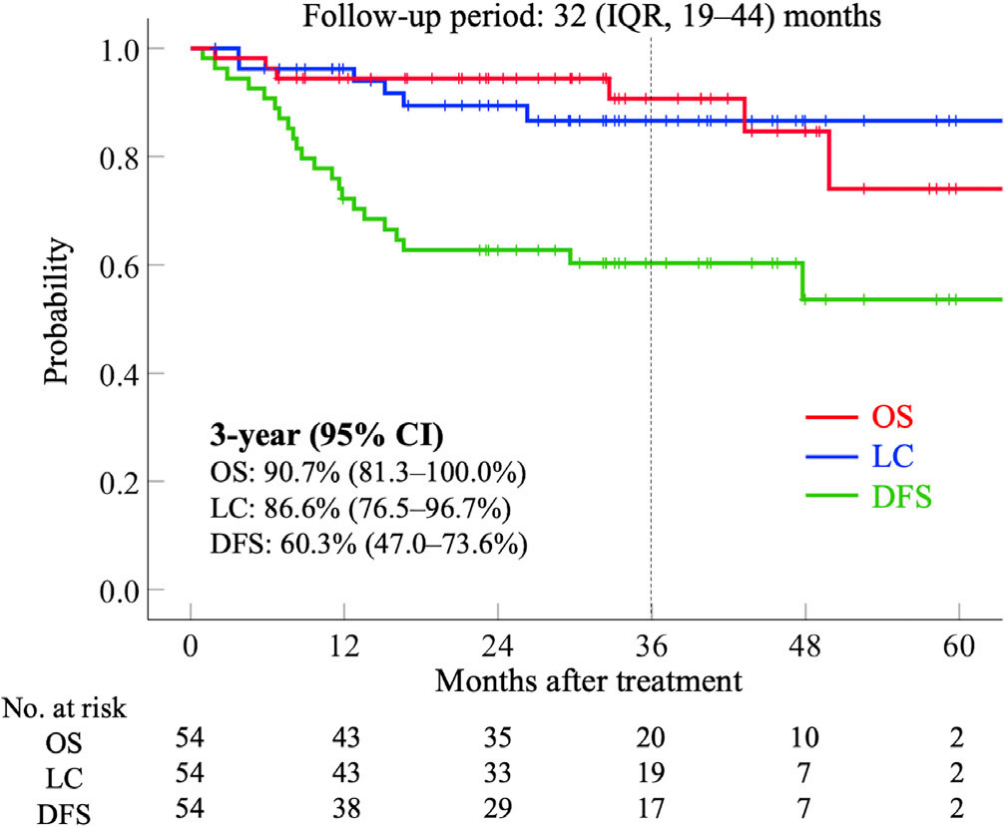
Clinical outcomes calculated by the Kaplan–Meier method. Red line indicates overall survival, blue line indicates local control, and green line indicates disease‐free survival. CI, confidence interval; DFS, disease‐free survival; IQR, interquartile range; LC, local control; No., number; OS, overall survival

No patient complained of symptoms such as persistent bleeding or abdominal pain after the treatment. Figure 2 shows the clinical outcomes of the entire cohort of the present study. The 3‐year LC, DFS, and OS rates for all 54 patients were 86.6% (95% confidence interval [CI] 76.5%–96.7%], 60.3% (95% CI [47.0%–73.6%]), and 90.7% (95% CI [81.3%–100.0%]), respectively. Of the 54 patients analyzed, 52 were still alive, and two patients had died at the last follow‐up. Local tumor recurrence was observed in six of the 54 patients by the last follow‐up. Of the six patients with local tumor recurrence, three had Sq, and the other three had Ad/Adsq. All six patients who had local recurrence had pelvic lymph node or distant metastatic recurrence at or shortly after the time of diagnosis of local recurrence. Of the six patients with local tumor recurrence, five received chemotherapy, and the remaining patient received supportive care due to old age and weakened general condition. Four had died of cancer, and two were still alive with the disease at the last follow‐up date.

Table 2 shows the results of the univariate analyses based on clinical factors. The 3‐year LC rate was 87.7% in patients with FIGO III–IVA and 90.4% in patients with tumor size >6.0 cm. The 3‐year LC rate for Sq was 92.6%, while the 3‐year LC rate for Ad/Adsq was 66.7%, with Ad/Adsq showing a slightly worse LC rate. No statistically significant differences were observed in LC classified by FIGO stage (IB–II vs. III–IVA), histological subtypes, tumor size (≤6.0 cm vs. >6.0 cm), and concurrent use of chemotherapy. No statistically significant differences were observed in DFS or OS according to these clinical factors.

**TABLE 2:** Univariate analyses by clinical factors

Factor	No. of patients	LC		DFS		OS	
		3‐year (%)	*p* value	3‐year (%)	*p* value	3‐year (%)	*p* value
FIGO stage (2008)			.955		.474		.356
IB–II	26	86.0		65.4		89.3	
III–IVA	28	87.7		54.5		92.9	
Histological subtypes			.053		.551		.889
Sq	45	92.6		58.9		93.3	
Ad/Adsq	9	66.7		66.7		83.3	
Tumor size			.600		.241		.938
≤6.0 cm	31	83.6		54.4		90.7	
>6.0 cm	23	90.4		69.1		91.3	
Concurrent chemotherapy			.257		.568		.401
No	15	79.0		66.7		93.3	
Yes	39	90.2		58.2		89.9	

Abbreviations: Ad, adenocarcinoma; Adsq, adenosquamous carcinoma; DFS, disease‐free survival; FIGO, International Federation of Gynecology and Obstetrics; LC, local control; No., number; OS, overall survival; Sq, squamous cell carcinoma.

There were no adverse events related to the CT‐based transvaginal HBT procedure. Regarding late rectal toxicities, five patients developed grade ≥1 rectal toxicity. Two patients developed grade 1 toxicity, and three patients developed grade 2 toxicity requiring blood transfusion, argon plasma coagulation, or short‐term systemic corticosteroid use. The patient who required corticosteroids had been using 0.5 mg of dexamethasone tablet for about 4 weeks. None of the patients developed grade ≥3 rectal toxicity. Regarding late bladder toxicities, six patients developed grade ≥1 bladder toxicity, four patients developed grade 1 toxicity, and one patient developed grade 2 toxicity requiring blood transfusion. One patient developed grade 4 toxicity and a vesicovaginal fistula. The patient who developed grade 4 toxicity originally presented with a bladder‐invading tumor. Thus, the incidence of late adverse events of grade ≥3 in the rectum and bladder was 0% and 1.8%, respectively.

### 3.3 Relationship between dose‐volume histogram parameters and clinical outcomes

Table 3 shows the relationship between dose‐volume histogram (DVH) parameters and clinical outcomes. Regarding the cumulative dose of HR‐CTV_D90_, the mean ± SD values of brachytherapy in patients without and with local recurrence were 41.5 ± 5.1 Gy EQD_2_ and 38.9 ± 7.1 Gy EQD_2,_ respectively, with no statistically significant differences observed (*p* = .336). The mean dose of WP was significantly higher in patients with local recurrence (*p* = .022); three of the six patients had received WP up to the equivalent of 40 Gy because the shrinkage of the tumor was slow in these three cases. The combined dose of WP and brachytherapy was not significantly different between the groups (*p* = .572) (Table 3A).

**TABLE 3:** Relationship between dose and local control/late adverse events

(A) Dose of HR‐CTV and local control/recurrence			
Factor	HR‐CTV_D90_ (EQD2): mean ± SD		
	BT (Gy)	WP (Gy)	BT + WP (Gy)
Local controlled (*n* = 48)	41.5 ± 5.1	30.4 ± 3.5	71.9 ± 4.8
Local recurrence (*n* = 6)	38.9 ± 7.1	34.2 ± 4.7	73.1 ± 4.6
*p* value	.277	.022	.572

(B) Dose of rectum D_2cc_ and late rectal toxicity			
Factor	Rectum D_2cc_ (EQD2): mean ± SD		
	BT (Gy)	WP (Gy)	BT + WP (Gy)
Rectum grade 0 (*n* = 49)	34.0 ± 8.0	30.4 ± 3.8	64.4 ± 9.2
Rectum grade ≥1 (*n* = 5)	37.4 ± 7.2	31.5 ± 4.8	68.9 ± 6.7
*p* value	.374	.558	.301

(C) Dose of bladder D_2cc_ and late bladder toxicity			
Factor	Bladder D_2cc_ (EQD2): mean ± SD		
	BT (Gy)	WP (Gy)	BT + WP (Gy)
Rectum grade 0 (*n* = 48)	43.1 ± 9.8	30.6 ± 4.0	73.7 ± 11.3
Rectum grade ≥1 (*n* = 6)	47.9 ± 6.8	29.5 ± 0.3	77.4 ± 6.9
*p* value	.258	.512	.445

Abbreviations: BT, brachytherapy; EQD2, equivalent dose of 2 Gy per fraction; HR‐CTV, high‐risk clinical target volume; WP, whole pelvic irradiation.

Regarding the cumulative dose of rectum D_2cc_, the average ± SD values of brachytherapy in patients without and with toxicity were 34.0 ± 8.0 Gy EQD_2_ and 37.4 ± 7.2 Gy EQD_2,_ respectively. There were no statistically significant differences in the mean dose of rectum D_2cc_ between patients with and without toxicity in any classification, brachytherapy, WP, or brachytherapy plus WP (Table 3B). Regarding the cumulative dose of bladder D_2cc_, the average ± SD values of brachytherapy in patients without and with toxicity were 43.1 ± 9.8 Gy EQD_2_ and 47.9 ± 6.8 Gy EQD_2,_ respectively. There were no statistically significant differences in the mean dose of bladder D_2cc_ between patients with and without toxicity in any classification, brachytherapy, WP, or brachytherapy plus WP (Table 3C).

### 3.4 Timing and significance of HBT

For all 54 patients analyzed in the present study, a total of 215 brachytherapy sessions were performed, including conventional brachytherapy, which did not use interstitial catheters, and transvaginal HBT. Table 4 shows the ratio of conventional brachytherapy to transvaginal HBT in each session. Five sessions of brachytherapy were performed on one patient. Except for the fifth session of brachytherapy, transvaginal HBT was performed most frequently in the second session.

**TABLE 4:** Ratio of conventional brachytherapy and hybrid brachytherapy in each session

	First session	Second session	Third session	Fourth session	Fifth session
Conventional, *n* (%)	17 (31.5)	12 (22.2)	16 (29.6)	15 (28.8)	0 (0.0)
Hybrid, *n* (%)	37 (68.5)	42 (77.8)	38 (70.4)	37 (71.2)	1 (100.0)

Abbreviation: n, number.

Next, we compared the dose parameters in HR‐CTV and OARs between conventional brachytherapy and transvaginal HBT (Table 5). The mean dose (range) of HR‐CTV_D90_ in conventional brachytherapy and transvaginal HBT were 6.81 (3.89–8.47) Gy and 7.32 (3.41–9.89) Gy, respectively. The mean dose of HR‐CTV_D90_ in transvaginal HBT was significantly higher than that in conventional brachytherapy (*p* < .001). The mean dose (range) of rectum D_2cc_ in conventional brachytherapy and transvaginal HBT were 5.09 (2.18–7.18) Gy and 5.21 (2.39–7.93) Gy, respectively. The mean dose (range) of bladder D_2cc_ in conventional brachytherapy and transvaginal HBT were 5.99 (4.28–8.24) Gy and 5.99 (3.49–10.35) Gy, respectively. There were no statistically significant differences between conventional brachytherapy and transvaginal HBT with respect to rectum D_2cc_ and bladder D_2cc_.

**TABLE 5:** Comparisons of dose parameters in HR‐CTV and OARs between conventional and hybrid brachytherapy

	Conventional (Gy), 60 sessions	Hybrid (Gy), 155 sessions	*p* value
HR‐CTV_D90_, range (mean)	3.89–8.47 (6.81)	3.41–9.89 (7.32)	<.001
Rectum D_2cc_, range (mean)	2.18–7.18 (5.09)	2.39–7.93 (5.21)	.416
Bladder D_2cc_, range (mean)	4.28–8.24 (5.99)	3.49–10.35 (5.99)	1.000

Abbreviations: HR‐CTV, high‐risk clinical target volume; OARs, organs at risk.

We further assessed the difference in the incidence of local recurrence among patients who did and did not receive HBT at the first session. Of the 37 patients who received HBT at the first session, four had local recurrence. In contrast, of the 17 who did not receive HBT at the first session, two had local recurrence. There was no statistically significant difference in the frequency of recurrence between the two groups (*p* = .917). Regarding the total dose to the HR‐CTV_D90_, the group that received HBT for the first time had a significantly higher total dose (~4.8% [72.0 ± 4.8 Gy EQD_2_ vs. 68.7 ± 4.2 Gy EQD_2_; *p* = .020]) compared to the group that did not receive HBT at the first session. However, there were no statistically significant differences in the mean dose of rectum D_2cc_ or bladder D_2cc_ between the two groups (Table 6).

**TABLE 6:** Comparisons of incidence of local recurrence, dose parameters in HR‐CTV and OARs between patients who missed the HBT at the first session and those who received HBT at the first session

	No. of patients with local recurrence	HR‐CTV_D90_ (EQD2): mean ± SD BT + WP	Rectum D_2cc_ (EQD2): mean ± SD BT + WP	Bladder D_2cc_ (EQD2): mean ± SD BT + WP
Patients who received the HBT at the first session (*n* = 37)	4	72.0 ± 4.8	64.9 ± 8.9	74.1 ± 11.0
Patients who missed the HBT at the first session (*n* = 17)	2	68.7 ± 4.2	61.4 ± 9.4	72.0 ± 8.2
*p* value	.917	.020	.201	.494

Abbreviations: BT, brachytherapy; HBT, hybrid brachytherapy; high‐risk clinical target volume; No., number; OARs, organs at risk; WP, whole pelvic irradiation.

## 4 DISCUSSION

First, we found favorable clinical outcomes, especially in LC rate in the present study. Previous studies consisting of non‐IGBT suggested that tumor size and stage were poor prognostic factors for cervical cancer.^30^, ^31^ Parker et al. reported that the 2‐years LC rate in tumors sized >6 cm was 51.9%.^31^ In contrast, the overall 3‐year LC rate was 86.6%. Notably, the 3‐year LC rate in the present study was 90.4% in patients with tumor size >6.0 cm. This improvement in LC is attributed to the effect of IGBTs, and the LC values are comparable to that reported in EMBRACE‐I, which was performed with MRI‐based IGBTs.^11^ The incidence of late adverse events of grade ≥3 in the rectum and bladder was 0% and 1.8%, respectively. Therefore, CT‐based hybrid brachytherapy for cervical cancer may be a reasonable treatment strategy for this disease.

In the present study, we did not find any relationship between DVH parameters and clinical outcomes. This was probably due to the small number of local recurrences and late adverse events. Because a combination of WP irradiation and CS was used in the present study, interpreting the values of DVH parameters requires close attention. Among studies that used a similar method, including WP irradiation and CS, Okazaki et al. reported that HR‐CTV_D90_ and HR‐CTV_D98_ doses for brachytherapy sessions were significantly associated with LC.^20^ In their analysis, it was demonstrated that the aiming dose of HR‐CTV_D90_ for brachytherapy sessions to achieve favorable LC is 36.0 EQD_2_. The mean ± SD values of HR‐CTV_D90_ for brachytherapy sessions was 41.5 ± 5.1 Gy EQD_2_ in patients with local controlled cancer in the present study. Thus, our results support the findings of Okazaki et al.^20^ However, the mean ± SD values of HR‐CTV_D90_ for brachytherapy sessions were 38.9 ± 7.1 Gy EQD_2_ in the patients with local recurrence, and the dose to the HR‐CTV_D90_ itself did not appear to be insufficient even in the recurrent cases. It may be noteworthy that three of the six cases with local recurrence were Ad/Adsq subtypes. In fact, many recent reports indicate that patients with Ad/Adsq of cervical cancer show lower LC rates compared to those with the Sq subtype.^32^, ^33^, ^34^ Further studies are needed to determine whether there is indeed a difference in the dose required for local control of Ad/Adsq and Sq, and if so, the optimal dose to cure Ad/Adsq.

One of the clinical utilities of CT‐based transvaginal HBT is that it does not require a saddle block or general anesthesia. No patient complained of symptoms such as persistent bleeding or abdominal pain after the treatment. Moreover, there were no adverse events related to CT‐based transvaginal HBT with intravenous sedation in the present study. Another convenience of CT‐based transvaginal HBT is the easy switching from conventional brachytherapy to HBT during the procedure. As mentioned above, CT‐based transvaginal HBT does not require general anesthesia or a special applicator. In fact, transvaginal HBT was performed most frequently during the second session in the present study. A similar tendency was also reported in a recent dosimetric survey for CT‐based IGBT, including HBT, in a Japanese prospective multi‐institutional study.^35^ This is probably the result of switching to HBT in the second session when an insufficient dose of HR‐CTV or an excessive dose of OARs was observed in the first session using conventional brachytherapy. However, as shown in Table 6, patients who received the HBT at the first session obtained a higher dose of ~4.8% to HR‐CTV_D90_ than those who did not receive the HBT at the first session. There was no statistically significant difference in the frequency of recurrence between the two groups; this could be due to differences in background factors, particularly tumor size, between the two groups. Although LC advantage was not shown in this study if HBT was applied from the first session, we can obtain better HR‐CTV_D90_ without increasing doses to OARs; HBT should be performed from the first session for patients who need it.

Importantly, we used multiple analgesics with transvenous sedation in the present study. In addition, we always need to fully explain other treatment options, including anesthesia methods, to patients in any environment. With careful considerations of these points, CT‐based transvaginal HBT without performing general anesthesia or saddle block would be a good treatment option.

The present study also demonstrated the dosimetric advantage of HBT. Transvaginal HBT increased the dose of HR‐CTV_D90_ by about 7.5% (6.81 Gy for conventional brachytherapy and 7.32 Gy for HBT) without significantly increasing the dose of OARs. Liu et al. previously reported that CT‐based HBT is effective in cases with tumor diameters greater than 5 cm, using DVH analysis.^36^ In our study, the median tumor size was 6.0 cm, which is consistent with the results of Liu et al.^36^ Considering the favorable clinical results of our study, patients with a larger tumor size are good candidates for HBT. HBT can be applied not only to increase the dose to the HR‐CTV, but also to reduce the dose to the OARs. This perspective may be important, especially for irregularly shaped tumors. As a measure of this irregularity, we used the ellipticity in the present study, which was 2.07 (IRQ, 1.63–2.75). There have been no reports on the validation of appropriate ellipticity as an indication criterion for HBT. Although we have used certain criteria for HBT indications, further studies on these indications are necessary.

To date, many studies have introduced a combination of intracavitary and interstitial brachytherapy.^23^, ^25^, ^37^, ^38^ Interstitial ring applicators (Elekta Brachytherapy, Netherlands) and Venezia applicator (Elekta Brachytherapy, Netherlands) are already commercially available, and their clinical use is gradually increasing, especially in Europe and the United States. There is no doubt that these applicators are a logical way to combine intracavitary and interstitial brachytherapy. However, their high cost limits their use in hospitals in regions such as Asia and Africa, where the number of patients with cervical cancer is increasing.^16^ The method we have reported here could be implemented with existing applicators and thus can be expected to be implemented in more facilities.

This study has several limitations which include the small cohort of patients and short follow‐up periods. The lack of significant differences in DFS and OS in stage and tumor size is probably due to these limitations. In addition, this was a single‐institution retrospective analysis. A multicenter prospective study is currently ongoing to determine the clinical significance of CT‐based transvaginal HBT, and this study would validate our findings. Since the present study employed CT‐based IGBT and EBRT, including CS, sufficient care should be taken when comparing DVH parameters with studies employing MRI‐based IGBT and EBRT without CS. Furthermore, studies that actually compare cost‐effectiveness in our strategy with other MRI‐based IGBTs are needed.

In conclusion, we reported the clinical advantages of CT‐based transvaginal HBT. With the fact that none of the patients complained of symptoms after the procedure, favorable LC rate, mild toxicity, and possible cost‐effectiveness of the series of procedures, our strategy may be a good option for cervical cancer.

## CONFLICT OF INTEREST

The authors have stated explicitly that there are no conflicts of interest in connection with this article.

## AUTHOR CONTRIBUTIONS

**Noriyuki Okonogi:** Conceptualization (lead); data curation (equal); formal analysis (lead); investigation (equal); methodology (equal); visualization (lead); writing – original draft (lead). **Kazutoshi Murata:** Conceptualization (equal); formal analysis (equal); methodology (equal); writing – original draft (supporting). **Toshiaki Matsui:** Data curation (equal); investigation (equal). **Yuma Iwai:** Data curation (equal). **Yasumasa Mori:** Data curation (equal). **Takashi Kaneko:** Data curation (equal). **Masaru Wakatsuki:** Conceptualization (equal); formal analysis (equal); methodology (equal); writing – review and editing (equal). **Hiroshi Tsuji:** Writing – review and editing (equal).

## ETHICS STATEMENT

The study was approved by the Ethical Review Board committee of our institution (QST 20‐043, approved on March 11, 2021). Because of the retrospective nature, the need for written informed consent was waived for this study. Instead, patients who refused to be included in this study were given an opt‐out policy, which was uploaded on the webpage of our institution. The present study complied with the Declaration of Helsinki.

## Supporting information


**APPENDIX DATA S1** The specific examples for pain relief and sedation for brachytherapy.

**APPENDIX DATA S2** One of the criteria for implement of HBT in our hospital; The ellipticity

## Data Availability

Research data are stored in an institutional repository and will be shared upon request to the corresponding author.
