# Supplementary figures and images for: Clinical advantage and outcomes of computed tomography‐based transvaginal hybrid brachytherapy performed only by sedation without general or saddle block anesthesia
Source: Cancer Rep (Hoboken). 2022 Mar 1;5(11):e1607. doi: 10.1002/cnr2.1607 (PMC9675380; doi:10.1002/cnr2.1607)

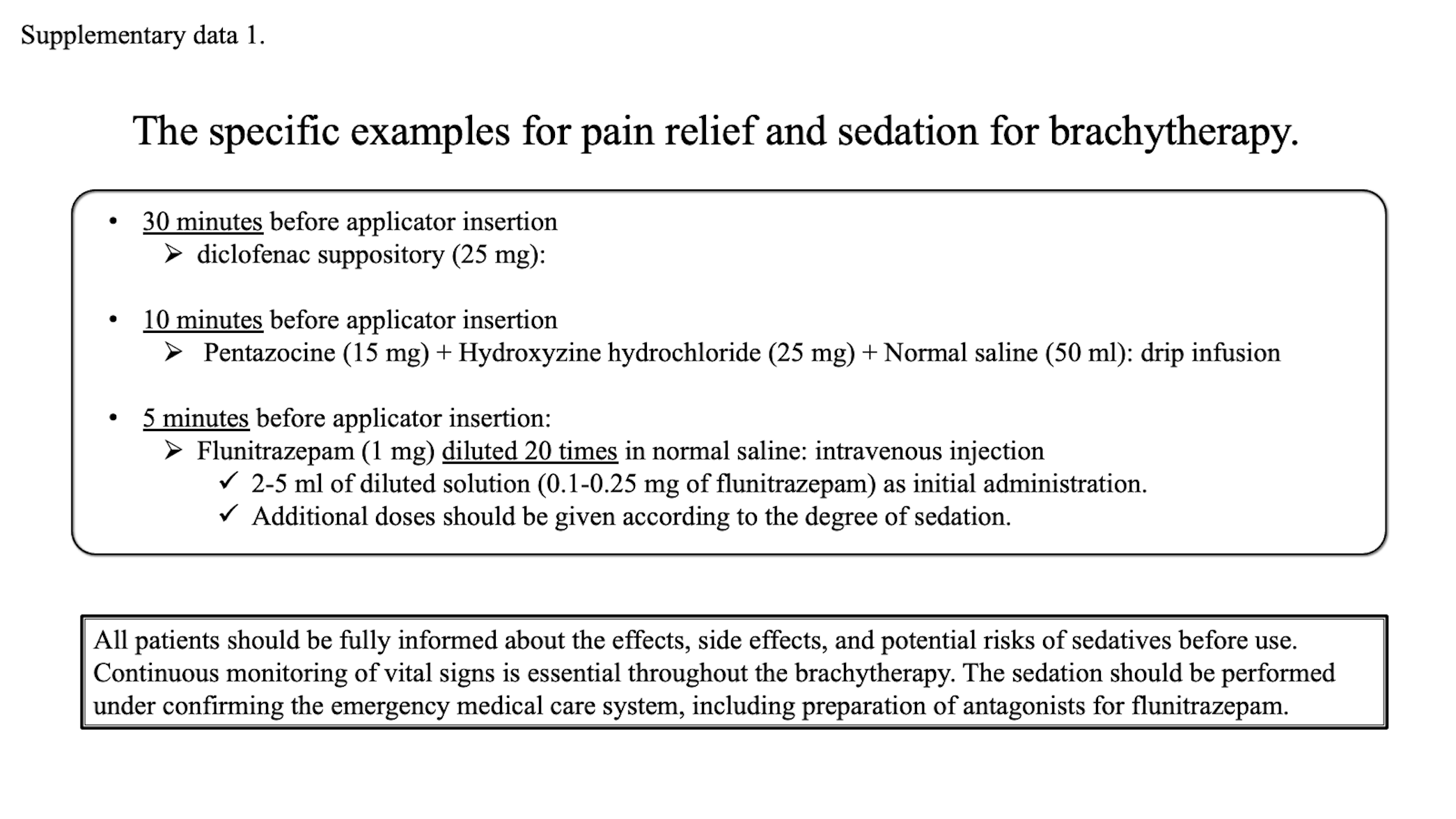

Supplement: Supplementary file 1 — APPENDIX DATA S1 The specific examples for pain relief and sedation for brachytherapy. [file CNR2-5-e1607-s001.tiff]

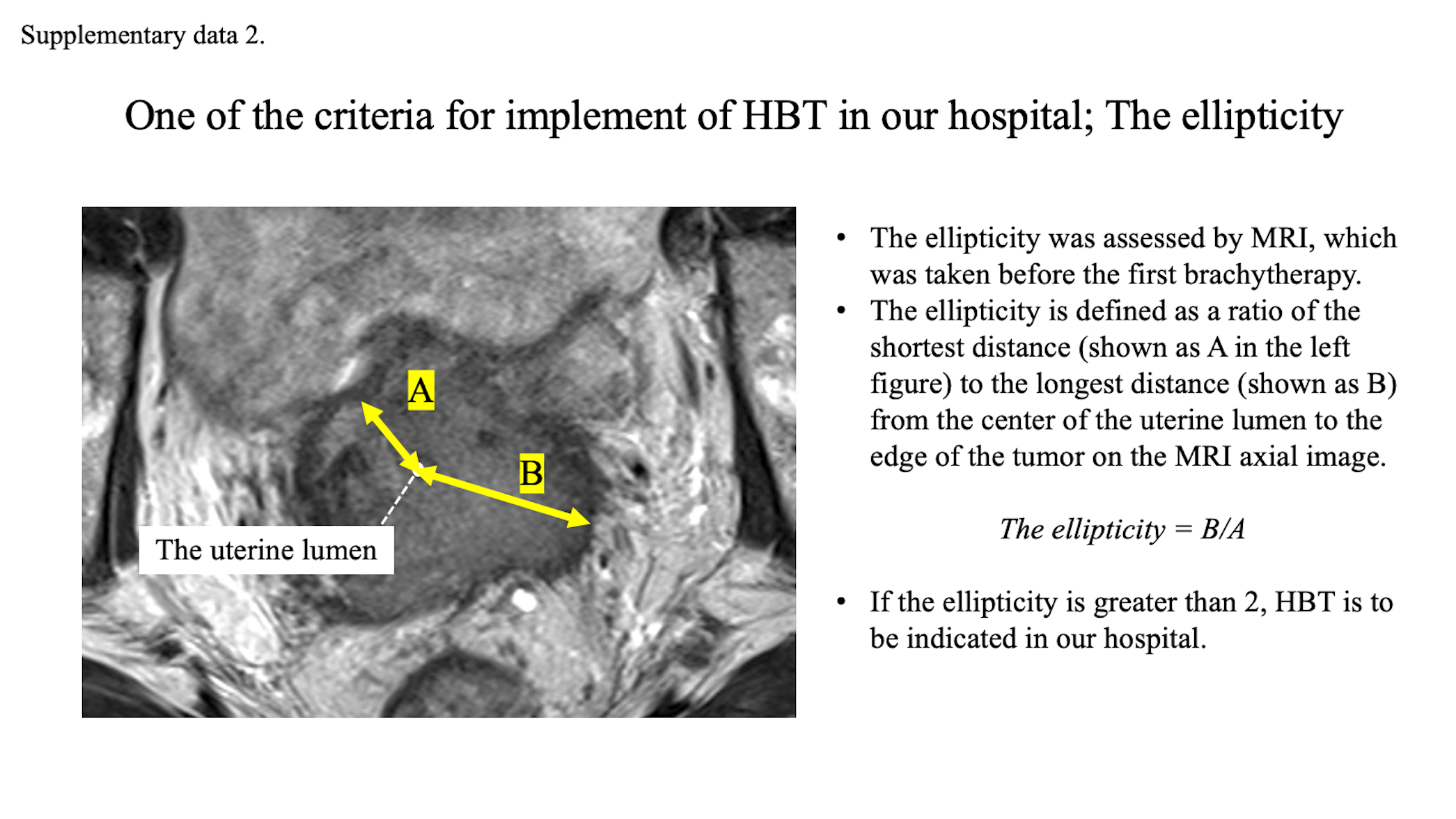

Supplement: Supplementary file 2 — APPENDIX DATA S2 One of the criteria for implement of HBT in our hospital; The ellipticity [file CNR2-5-e1607-s002.tiff]
